# Accurate and non-invasive localization of multi-focal ground-glass opacities via electromagnetic navigation bronchoscopy assisting video-assisted thoracoscopic surgery: a single-center study

**DOI:** 10.3389/fonc.2023.1255937

**Published:** 2023-10-23

**Authors:** Chenxi Zeng, Guang Yang, Lu Wei, Junhui Wang, Xue Wang, Fan Ye, Xiangning Fu, Yixin Cai, Jianing Wang

**Affiliations:** Department of Thoracic Surgery, Tongji Hospital, Tongji Medical College, Huazhong University of Science and Technology, Wuhan, China

**Keywords:** electromagnetic navigation bronchoscopy, multifocal ground glass opacities, localization, video-assisted thoracoscopic surgery, accuracy and safety

## Abstract

**Background:**

Accurate localization of multi-focal ground-glass opacities (GGOs) is crucial for successful video-assisted thoracoscopic surgery (VATS). Electromagnetic navigation bronchoscopy (ENB) provides a minimally invasive and dependable approach for precise localization. This study assessed the accuracy and safety of ENB-guided localization in cases involving multi-focal GGOs.

**Methods:**

This retrospective study presents a single-center investigation into ENB-guided localization, utilizing methylene blue, for multi-focal GGOs assisting VATS. Clinical, surgical, and pathological data were collected from patients who underwent ENB-guided localization between 23 December 2019 and 31 August 2022.

**Results:**

The study examined 57 patients with multi-focal GGOs who underwent ENB-guided localization and VATS. A total of 150 GGOs were treated, with ENB-guided localization taking a median time of 65 min. Following localization, all patients proceeded to VATS, with a median duration of 170 min. The median lesion size measured 7.8 mm, with a 5-mm distance between GGO and pleura or fissure. When the distance between GGO and pleura/fissure exceeded 1 cm, an additional location point was introduced below the pleura or fissure based on GGO location. No complications related to localization were observed. The overall malignancy rate stood at 66%. Location precision was confirmed by measuring the marker-to-GGO lesion distance, resulting in a 94% (141/150) accuracy rate for GGO localization.

**Conclusion:**

ENB-guided methylene blue injection is a safe and precise method to treat multi-focal GGOs, potentially minimizing operation time and simplifying lesion detection.

## Introduction

With the development of high-resolution chest CT, it has become evident that a large population of patients is being diagnosed with multi-focal ground-glass opacities (GGOs) (1). The presence of a GGO component in multiple pulmonary sites indicates a synchronous primary non–small-cell lung cancer (NSCLC) (2–4). Currently, there are no guidelines for diagnosing and treatment for multi-focal GGO, and study findings remain conflicting among various centers. Because no standard criteria for selecting opacities for treatment have been established, the question of whether only the predominant lesion should be treated remains controversial, especially because some studies have attributed survival to a simultaneous resection of “additional” nodules (3, 5, 6). In light of such findings, although difficult to achieve, precise localization of multi-focal GGO becomes a crucial part of thoracoscopic surgery (7), and all the more so because thoracic surgery remains the most effective treatment for pulmonary lesions.

Preoperative localization of pulmonary nodules, especially GGO, has been widely used in clinical practice, for video-assisted thoracic surgery in particular (8). The use of CT-guided localization has increased in popularity due to numerous advantages, such as cost-effectiveness, time efficiency, operating simplicity, and high success rate (9–12). However, there are also shortcomings of the procedure such as hemorrhage, pneumothorax, severe pain, pleural reaction, or needle-track implantation (13). The most critical limitation of CT-guided localization is the limited scan range possible with minimal invasiveness, especially when the location of multi-focal lesions needs to be identified during one operation. Compared with CT-guided localization, electromagnetic navigation bronchoscopy (ENB), an emerging diagnostic technology, enables more accurate localization of multiple nodules. ENB can reach all regions with almost no complications (14). During the ENB-guided localization, patients have no need to change their body position, which makes this procedure especially advantageous in treating multiple pulmonary nodules (15). In this study, we collected clinical and pathological data about patients with multi-focal GGO undergoing ENB-guided localization combined with VATS in the thoracic surgery department of Tongji Hospital. The next stage of the study was gathering the patients’ results after the procedure, including the data on the postoperative complication rate. The analysis of the two sets of data allowed us to evaluate both the safety and efficacy of ENB in the localization of multi-focal GGOs.

## Methods

### Patients and procedures

This study was approved by the Ethics Committee of Tongji Medical College of Huazhong University of Science and Technology (approval number 2019-S110-1; approval date, 26 May 2021). Informed consent was waived because of the retrospective nature of the study. We retrospectively collected clinical data from consecutive patients with multi-focal GGO who had undergone ENB-guided localization combined with VATS in the Thoracic Surgery Department of Tongji Hospital from 23 December 2019 to 31 August 2022. Inclusion criteria were as follows: more than two GGOs on chest CT; the predominant lesion, with highly suspicious imaging signs of malignancy, should fulfill either of the following criteria: i. diameter exceeding 10 mm for pure GGO and ii. no size alteration or growth over a minimum of 3 months; residual GGOs’ diameter over 6 mm; without any intra-pulmonary or organ metastasis evidences and surgical candidacy; agreement to receive ENB-guided localization; more than two GGOs located using ENB and then resected using VATS. Clinical, radiographic, surgical, and pathological data were collected. Details on a located GGO including its size, distance to the pleura, time for localization, accuracy of localization, and pathological diagnosis were obtained for analysis.

**Figure 1:**
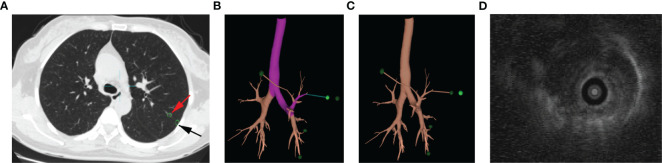
ENB-guided MWA procedure: targeting nodule in the chest CT **(A)**, red arrow points to the GGO, and black arrow points to the subpleural point of the GGO; planning of targeting GGO **(B)**; subpleural point added along the airway route **(C)**; EBUS-GS image of nodule **(D)**.

All ENB procedures used the superDimensionTM navigation system version 7.0 with manufacture’s instruction. Patients underwent chest CT within 1 week before surgery (**Figure 1A**). Virtual airway routes to the lesions were generated from preprocedural CT using the navigation platform to locate the given GGO. In case when the distance between GGO and the pleura was more than 1c m, an additional subpleural point was added along the airway route (**Figures 1B, C**). The ENB was performed under general anesthesia with laryngeal mask airway (LMA). After accessing the target lesion, endobronchial ultrasonography with a guide sheath (EBUS-GS) was used to confirm the location of the GGO (**Figure 1D**). Then 0.2–0.3mL of methylthionine chloride was injected into the catheter to mark each lesion including the additional subpleural points. After the procedure, LMA was replaced with a double-lumen endotracheal tube, and unilateral or bilateral uni-portal VATS was performed to resect the located GGO.

Shortly after endotracheal tube replacement, the patient was placed in the left or right lateral decubitus position. Surgical procedure was performed through a 3-cm single port, as we have previously mentioned (16). The location of lesions for resection was visually detected on the pleura as blue staining. To verify whether the locations were precisely marked, the resected GGOs were cut open. Within the imprecisely marked GGOs, a distance between the lesion and the blue staining was measured. Finally, a postoperative histopathological assessment of each resected GGO was carried out.

### Statistical analysis

Values are expressed as median (range) for continuous variables and as the total number (percentage) for categorical variables. All statistical analysis was performed using SPSS (version 27).

## Result

### Patient characteristics

A total of 57 patients received ENB-guided localization and VATs from 23 December 2019 to 31 August 2022. In this cohort, all 57 patients had multiple GGOs, and more than two GGOs were resected in each case under the localization of ENB. Patient characteristics are shown in **Table 1**. Patient details are outlined in **Table 1**. Median age stood at 52 years (31–73), with 48 patients being women. The interval between surgery and initial GGO detection averaged 150 days (30–1,460).

**Table 1:** Summary of patients’ characteristics.

		Counts (%)	Median	Range
Age			52	31–73
Sex				
	Male	9 (15.8)		
	Female	48 (84.2)		
Smoking				
	Yes	4 (16.9)		
	No	53 (83.1)		
Drinking				
	Yes	1 (3.1)		
	No	56 (96.9)		
Family history				
	Yes	11 (21.5)		
	No	46 (78.5)		
COPD				
	Mild	2 (9.2)		
	No	55 (89.2)		
Lung function				
	FEV1		2.43	1.26–3.89
	FEV1%		102.4	83.4–139.4
	FEV1/FVC ratio		0.75	0.65–0.90
CEA			1.25	0.5–4.12
NSE			12.62	8.63–24.33
CYFRA21-1			1.98	0.81–6.30
SCC			0.70	0.3–2.70
proGRP			40.9	13.55–77.6
VATs time (min)			170	70–320

COPD, chronic obstructive pulmonary disease; CEA, carcinoembryonic antigen; NSE, neuron-specific enolase; CYFRA21-1, cytokeratin 19 fragment; SCC, small-cell carcinoma; proGRP, progastrin-releasing peptide.

### Nodule characteristics and pathological results

Information on the nodules is shown in **Table 2**. A total of 150 nodules in 57 patients were resected. A total of 150 nodules were located preoperatively using ENB. All the nodules were GGOs. The median diameter of GGOs was 7.8 (4.2–21) mm. The median distance between nodules and the pleura or the pleural fissure was 5 mm (0–29). A total of 119 nodules were deep-seated (the distance to the pleura or the pleural fissure was between 1 cm and 3 cm). Median ENB procedure time per patient was 65 (40–120) min. Median VATS time per patient was 170 (70–320) min. A total of 114 nodules were removed by a wedge resection. Twenty-one nodules were removed by segmentectomy. Thirteen nodules were removed by combined segmentectomy. In addition, two nodules were removed by lobectomy.

**Table 2:** Characteristics of nodules treated by VATS combined with ENB-guided localization.

Nodules located via ENB (n = 150)			Counts (%)	Median	Range
Nodule size (mm)				7.8	6–21
Pulmonary segments	Right	SI	15 (10.0)		
		SII	21 (14.0)		
		SIII	14 (9.3)		
		SIV-SV	13 (8.7)		
		SVI	8 (5.3)		
		SVII-SX	21 (14.0)		
	Left	SI+II	16 (10.6)		
		SIII	10 (6.7)		
		SIV-SV	8 (5.3)		
		SVI	10 (6.7)		
		SVII-SIX	14 (9.3)		
Distance to pleura or fissure				5.0	0–29
	Less than 1 cm		31 (20.7)		
	Between 1 cm and 3 cm		119 (79.3)		
Type	Ground-glass opacity		150 (100.0)		
Surgery	Wedge resection		114 (76.0)		
	Segmentectomy		21 (14.0)		
	Combined segmentectomy		13 (8.7)		
	Lobectomy		2 (1.3)		

Pathological results are shown in **Table 3**. Postoperative pathology was performed on each resected nodule. The overall malignancy of nodules was 66%. There were 14 cases of invasive adenocarcinoma, 36 of minimally invasive adenocarcinoma, 49 of adenocarcinoma *in situ*, 23 of atypia, and 28 were benign lesion or negative for malignancy.

**Table 3:** Pathology of nodules.

Nodules treated by VATS (n = 150)	Counts (%)
Adenocarcinoma	14 (9.3)
MIA	36 (24.0)
AIS	49 (32.7)
Atypia	23 (15.3)
Chronic granuloma	6 (4.0)
MPMNs	1 (0.7)
Negative for malignancy	21 (14.0)

MIA, minimally invasive adenocarcinoma; AIS, adenocarcinoma in situ; MPMNs, minute pulmonary meningothelial-like nodules.

### Complications and localization accuracy

Information on postoperative complication is shown in **Table 4**. Localization procedures were performed on all patients under general anesthesia. Hence, differing from CT-guided localization, patients did not encounter post-procedural pain, pleural reactions, subcutaneous emphysema, or hemorrhage resulting from a thoracic incision. Moreover, there were no instances of pneumothorax or hemothorax following ENB localization.

**Table 4:** Complications after ENB-guided localization.

Complications	Counts
Persistent pain	0
Pleural reaction	0
Subcutaneous emphysema	0
Hemorrhage	0
Pneumothorax	0
Hemothorax	0

Each lesion was cut open to judge the accuracy of localization. When there was no overlap between the dye-mark and the lesion, mislocalization was assigned. Data on localization accuracy are shown in **Table 5**. The overall accuracy was 94% (141/150). Only nine GGOs were not stained by methylthionine chloride. Nevertheless, the maximum distance between dye-mark and lesion was 8 mm in these GGOs, of which six were in the lower lobe and three were in the apical segment of the upper lobe.

**Table 5:** ENB-guided localization accuracy.

Variables	Counts (%)
Dye-mark visualization on the lesion	141 (94)
Mislocalization	9 (6)
Lower lobe	6 (4)
Upper lobe (apical segment)	3 (2)
Maximum distance between dye-mark and lesion	8 mm

### A case of multi-focal GGOs receiving ENB-guided localization combined with VATs

**Figure 2:**
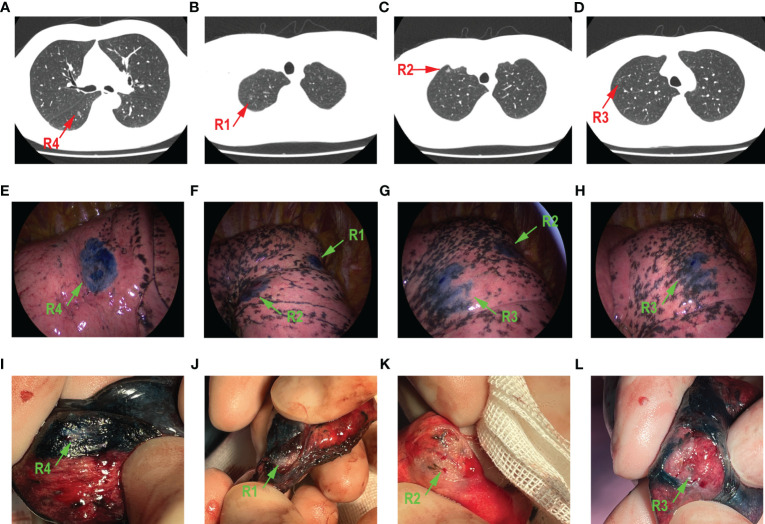
A 50-year-old male patient who had four GGOs in right lung. The main lesion R4 was in the anterior basal segment **(A)**. R1 **(B)** and R2 **(C)** were both in the apical segment. R3 **(D)** is in the anterior segment. The localization of methylthionine chloride under the thoracoscope is shown in **(E–H)**, respectively, green arrow points to a different lesion. All GGOs were removed through wedge resection. The resected lung tissue is shown in **(I–L)**, respectively; green arrow points to different lesion.

A 50-year-old patient was diagnosed with multi-focal GGOs in his right lung. **Figures 2A–E** show the preoperative enhanced chest CT of this patient. The predominant lesion was in the lower lobe of right lung (R4), in the anterior basal segment. The diameter of this GGO was 8 mm (**Figure 2A**). Moreover, he had other three GGOs in the right lung and two GGOs in the apical segment (R1 and R2) in the upper lobe, with the diameters 9 mm and 6.5 mm, respectively. The other one is in the anterior segment (R3), in the upper lobe, and its diameter was 6 mm (**Figures 2B–D**). The overall time of ENB procedure was 137 min. **Figures 2E–H** show the exact location of four localized GGOs under the thoracoscope. Wedge resection was performed in these four GGOs. The time of VATs was 160 min. **Figures 2I–L** show the resected lung tissue. As is shown, an overlap of methylthionine chloride and the lesion was observed, which confirmed the accuracy of ENB-guided localization. Postoperative pathological evaluation showed that R1, R2, and R3 were adenocarcinoma *in situ*, and R4 was a minimally invasive adenocarcinoma.

## Discussion

ENB-guided localization of multi-focal GGOs has been demonstrated as a safe and effective diagnostic technique, as recent studies suggest. Performing ENB during VATS yielded good diagnostic results in the preoperative phase, according to the study. ENB-guided localization was not only highly accurate (94%) but proved not to carry, so common in thoracic surgery, such severe complications as persistent pain, pleural reaction, subcutaneous emphysema, hemorrhage, pneumothorax, or hemothorax. Moreover, accurate localization of lesions enables the resection of each, even the deepest multi-focal GGOs. Our results suggest that ENB-guided localization combined with VATS could be a reliable treatment for multi-focal GGOs.

The rampant advancement of high-resolution CT has led to a great increase in the number of primary lung cancer cases diagnosed within the population (1). However, the diagnosis and treatment of multiple cases of primary lung cancer was difficult due to the presence of solid lesions. Distinguishing primary lesions from metastases of a primary lung cancer is of great importance in therapeutic strategy. Several studies claim that the presence of multi-focal GGOs indicates a synchronous primary NSCLC (17), but, in fact, exact criteria for the treatment of pulmonary multi-focal GGOs have been specified yet. The principal operative treatment that is being applied recommends the removal of the dominant lesions, based on radiological findings. It remains controversial whether to remove residual lesions. Shimada et al. (5) demonstrated that the postoperative survival of patients with multi-focal GGOs depends on the surgical management of the predominant lesion and that a blanket removal of all lesions did not change the prognosis for the patient. However, findings of other studies indicate that residual nodules should also be treated under the strategy of optimal surveillance, as this may influence the survival (3, 5, 6).

Identification and localization of GGOs are one of several challenges presented by the surgical treatment for patients with multi-focal lesions. Detecting and precisely pinpointing multi-focal GGOs can be intricate, particularly when they are small or widely distributed. Accurate localization is critical to the complete removal of pathological lesions and minimizing the risk of spreading to adjacent tissues (7). Various preoperative localization techniques have been devised to aid in identifying small lesions during VATS, including CT-guided percutaneous localization, and ENB-guided localization. Among these, CT-guided percutaneous localization has been associated with pneumothorax, noted as the most prevalent complication (12, 18). In addition, the incidence of pulmonary hemorrhage during CT-guided percutaneous localization has been documented to be around 18% (19). Moreover, the rate of dislodgement of the dye-mark can vary significantly, ranging from 12% to 33% (9, 18, 20). Furthermore, in the case of patients with multi-focal GGOs, various locations of lesions may require adjusting the patient’s position during the procedure. This may be both uncomfortable to the patient and inconvenient to the surgeon. Nevertheless, ENB-guided localization had a huge advantage in localizing multi-focal GGOs over the localization guided by CT. As a minimally invasive treatment, ENB is able to reach any position in the entire lung, without the necessity of adjusting the patient’s position. Furthermore, without involving the risk of complications resulting from a thoracic incision or from the exposure to radiation, ENB-guided localization is also a significantly safer technique (13, 21).

The inaccuracy rate of CT-guided localization ranges from 0% to 10.3%, according to several studies (12, 22–24). In this study, the accuracy of ENB-guided localization was 94%. Mariolo et al. (25) also reported the success rate as 94% in 48 nodules with the diameters less than 15 mm. Zhang et al. (26) reported the success rate as 98.3% in 181 nodules with mean diameters of 9.21 ± 4.81mm. In our study, the median diameter of nodules was only 7.8 mm, which increased the navigational error. A small lesion <1 cm causes the localization accuracy to fall sharply (27). We used EBUS-GS to confirm the location, which may increase the accuracy in very small GGOs. Some GGOs are difficult to locate during VATS if methylthionine chloride is injected into the lesion only when the distance between the GGO and the pleura or fissure is long, and methylthionine chloride cannot penetrate to the pleura. Hence, to provide a better vision for the surgeon, we added an additional subpleural point along the airway route, based on the specific GGO location. Adding this additional point enabled a wedge resection of localized GGOs, which helped to preserve more pulmonary tissue. Lesions located in the upper lobe or distant from the bronchus were more difficult to localize (26, 28). Our findings indicate that mislocalization happened more often in the lower lobe or in the apical segment of the upper lobe. It suggests that the volumetric change in lower lobe during respiration was higher than that in other lobes, because lower lobe was attached to the diaphragm. Previous studies also reported difficulties in the upper lobe or a lesion distant from the bronchus. High resolution of preoperative chest CT plays an important role in increasing the accuracy of localization resulting from the clear bronchus sign. Moreover, positive end-expiratory pressure ventilation (PEEP) is also recommended in the ENB-guided localization procedure, especially for lesions in the lower lobe, to reduce the volume change caused by respiration (29).

Overall, a highly supportive role of ENB-guided localization in uni-portal VATS is greatly corroborated by our results. The median time of VATS was only 170 min, for resecting multiple GGOs. We sharply reduced the time during which the surgeon could find the exact GGO for resection. Even in mislocated GGOs, surgeon usually spent less than 2 min on finding the specific lesion. Likewise, the aforementioned observation that multi-focal GGOs would indicate a synchronous primary NSCLC was substantiated by the results of the postoperative pathological evaluation in our study. The overall malignant rate in all 150 nodules was 66%.

Our findings seem to make a significant impact on the widely approved operative mode for multi-focal GGOs. To the best of our knowledge, this is the most extensive study to date that assesses preoperative ENB-guided localization of multi-focal GGOs combined with uni-portal VATS. There are also some limitations to our study. First, this is a retrospective study with inevitable intrinsic shortcomings. Second, the cost of ENB-guided localization is greater than that of CT-guided localization, which may be an inhibitory effect on promoting this technique in clinical practice. Third, although high-resolution chest CT may support ENB-guided localization to increase the accuracy, ENB may still mislocalize GGOs as many lesions do not have a CT bronchus sign. Hence, the whole procedure of ENB-guided localization should be performed carefully so that to increase the accuracy, which is crucial in uni-portal VATS. Although the findings of our study corroborated the theory of a useful role of ENB-guided localization during VATS, a further study carried out on more multi-focal GGO would confirm diagnostic value of using the two techniques simultaneously during one procedure.

## Conclusions

Our study demonstrated that ENB-guided localization of multi-focal GGOs has a great enhancing effect on the performance of uni-portal VATS, with no related complications. This is an effective technique to sharply reduce the operation time and to aid in the “blanket” resection and effective treatment for multi-focal GGOs as well as to make the accurate pathological evaluation of specific lesions. Our results are of promising clinical relevance and should be validated by a larger sample size. Further investigation and clinical practice are needed to increase the efficacy of ENB-guided localization of multi-focal GGOs during VATS.

## Data availability statement

The original contributions presented in the study are included in the article/supplementary material. Further inquiries can be directed to the corresponding authors.

## Ethics statement

The studies involving humans were approved by the ethic committee of Tongji Medical College of Huazhong University of Science and Technology. The studies were conducted in accordance with the local legislation and institutional requirements. The participants provided their written informed consent to participate in this study. Written informed consent was obtained from the individual(s) for the publication of any potentially identifiable images or data included in this article.

## Author contributions

CZ: Writing – original draft. GY: Writing – original draft. LW: Project administration, Writing – review & editing. JHW: Project administration, Writing – review & editing. XW: Data curation, Writing – review & editing. FY: Data curation, Writing – review & editing. XF: Project administration, Writing – review & editing. YC: Conceptualization, Writing – review & editing. JNW: Conceptualization, Writing – review & editing.

## References

[1] Rubin GD. Lung nodule and cancer detection in computed tomography screening. J Thorac Imaging (2015) 30:130–8. doi: 10.1097/RTI.0000000000000140 PMC4654704 25658477

[2] Vazquez M Carter D Brambilla E Gazdar A Noguchi M Travis WD. Solitary and multiple resected adenocarcinomas after CT screening for lung cancer: histopathologic features and their prognostic implications. Lung Cancer (2009) 64:148–54. doi: 10.1016/j.lungcan.2008.08.009 PMC2849638 18951650

[3] Hattori A Matsunaga T Takamochi K Oh S Suzuki K. Radiological classification of multiple lung cancers and the prognostic impact based on the presence of a ground glass opacity component on thin-section computed tomography. Lung Cancer (2017) 113:7–13. doi: 10.1016/j.lungcan.2017.09.001 29110852

[4] Matsunaga T Suzuki K Takamochi K Oh S. New simple radiological criteria proposed for multiple primary lung cancers. Jpn J Clin Oncol (2017) 47:1073–7. doi: 10.1093/jjco/hyx113 28973259

[5] Shimada Y Saji H Otani K Maehara S Maeda J Yoshida K. Survival of a surgical series of lung cancer patients with synchronous multiple ground-glass opacities, and the management of their residual lesions. Lung Cancer (2015) 88:174–80. doi: 10.1016/j.lungcan.2015.02.016 25758554

[6] Detterbeck FC Marom EM Arenberg DA Franklin WA Nicholson AG Travis WD. The IASLC lung cancer staging project: background data and proposals for the application of TNM staging rules to lung cancer presenting as multiple nodules with ground glass or lepidic features or a pneumonic type of involvement in the forthcoming eighth edition of the TNM classification. J Thorac Oncol (2016) 11:666–80. doi: 10.1016/j.jtho.2015.12.113 26940527

[7] Sortini D Feo C Maravegias K Carcoforo P Pozza E Liboni A. Intrathoracoscopic localization techniques. Rev literature Surg Endosc (2006) 20:1341–7. doi: 10.1007/s00464-005-0407-z 16703435

[8] Nakashima S Watanabe A Obama T Yamada G Takahashi H Higami T. Need for preoperative computed tomography-guided localization in video-assisted thoracoscopic surgery pulmonary resections of metastatic pulmonary nodules. Ann Thorac Surg (2010) 89:212–8. doi: 10.1016/j.athoracsur.2009.09.075 20103238

[9] Thaete FL Peterson MS Plunkett MB Ferson PF Keenan RJ Landreneau RJ. Computed tomography-guided wire localization of pulmonary lesions before thoracoscopic resection: results in 101 cases. J Thorac Imaging (1999) 14:90–8. doi: 10.1097/00005382-199904000-00004 10210479

[10] Paci M Annessi V Giovanardi F Ferrari G De Franco S Casali C. Preoperative localization of indeterminate pulmonary nodules before videothoracoscopic resection. Surg Endosc (2002) 16:509–11. doi: 10.1007/s00464-001-9014-9 11928038

[11] Ciriaco P Negri G Puglisi A Nicoletti R Del Maschio A Zannini P. Video-assisted thoracoscopic surgery for pulmonary nodules: rationale for preoperative computed tomography-guided hookwire localization. Eur J Cardiothorac Surg (2004) 25:429–33. doi: 10.1016/j.ejcts.2003.11.036 15019673

[12] Chen YR Yeow KM Lee JY Su IH Chu SY Lee CH. CT-guided hook wire localization of subpleural lung lesions for video-assisted thoracoscopic surgery (VATS). J Formos Med Assoc (2007) 106:911–8. doi: 10.1016/S0929-6646(08)60061-3 18063512

[13] Park CH Han K Hur J Lee SM Lee JW Hwang SH. Comparative effectiveness and safety of preoperative lung localization for pulmonary nodules: A systematic review and meta-analysis. Chest (2017) 151:316–28. doi: 10.1016/j.chest.2016.09.017 27717643

[14] Jiang S Liu X Chen J Ma H Xie F Sun J. A pilot study of the ultrathin cryoprobe in the diagnosis of peripheral pulmonary ground-glass opacity lesions. Transl Lung Cancer Res (2020) 9:1963–73. doi: 10.21037/tlcr-20-957 PMC7653104 33209616

[15] Yang Q Han K Lv S Li Q Sun X Feng X. Virtual navigation bronchoscopy-guided intraoperative indocyanine green localization in simultaneous surgery for multiple pulmonary nodules. Thorac Cancer (2022) 13:2879–89. doi: 10.1111/1759-7714.14633 PMC9575123 36058556

[16] Wang Q Cai YX Deng Y Fu SL Fu XN Zhang N. Modular 3-cm uniportal video-assisted thoracoscopic left upper lobectomy with systemic lymphadenectomy. J Thorac Dis (2016) 8:2264–8. doi: 10.21037/jtd.2016.03.15 PMC4999770 27621888

[17] Hattori A Takamochi K Oh S Suzuki K. Prognostic classification of multiple primary lung cancers based on a ground-glass opacity component. Ann Thorac Surg (2020) 109:420–7. doi: 10.1016/j.athoracsur.2019.09.008 31593656

[18] Chen S Zhou J Zhang J Hu H Luo X Zhang Y. Video-assisted thoracoscopic solitary pulmonary nodule resection after CT-guided hookwire localization: 43 cases report and literature review. Surg Endosc (2011) 25:1723–9. doi: 10.1007/s00464-010-1502-3 21181200

[19] Heerink WJ de Bock GH de Jonge GJ Groen HJ Vliegenthart R Oudkerk M. Complication rates of CT-guided transthoracic lung biopsy: meta-analysis. Eur Radiol (2017) 27:138–48. doi: 10.1007/s00330-016-4357-8 PMC5127875 27108299

[20] Gonfiotti A Davini F Vaggelli L De Francisci A Caldarella A Gigli PM. Thoracoscopic localization techniques for patients with solitary pulmonary nodule: hookwire versus radio-guided surgery. Eur J Cardiothorac Surg (2007) 32:843–7. doi: 10.1016/j.ejcts.2007.09.002 17913505

[21] Eberhardt R Anantham D Herth F Feller-Kopman D Ernst A. Electromagnetic navigation diagnostic bronchoscopy in peripheral lung lesions. Chest (2007) 131:1800–5. doi: 10.1378/chest.06-3016 17400670

[22] Li W Wang Y He X Li G Wang S Xu L. Combination of CT-guided hookwire localization and video-assisted thoracoscopic surgery for pulmonary nodular lesions: Analysis of 103 patients. Oncol Lett (2012) 4:824–8. doi: 10.3892/ol.2012.800 PMC3506590 23205107

[23] Pittet O Christodoulou M Pezzetta E Schmidt S Schnyder P Ris HB. Video-assisted thoracoscopic resection of a small pulmonary nodule after computed tomography-guided localization with a hook-wire system. Experience in 45 consecutive patients. World J Surg (2007) 31:575–8. doi: 10.1007/s00268-006-0343-7 17318707

[24] Klinkenberg TJ Dinjens L Wolf RFE van der Wekken AJ van de Wauwer C de Bock GH. CT-guided percutaneous hookwire localization increases the efficacy and safety of VATS for pulmonary nodules. J Surg Oncol (2017) 115:898–904. doi: 10.1002/jso.24589 28230245

[25] Mariolo AV Vieira T Stern JB Perrot L Caliandro R Escande R. Electromagnetic navigation bronchoscopy localization of lung nodules for thoracoscopic resection. J Thorac Dis (2021) 13:4371–7. doi: 10.21037/jtd-21-223 PMC8339756 34422363

[26] Zhang J He J Chen J Zhong Y He J Li S. Application of indocyanine green injection guided by electromagnetic navigation bronchoscopy in localization of pulmonary nodules. Transl Lung Cancer Res (2021) 10:4414–22. doi: 10.21037/tlcr-21-699 PMC8743531 35070751

[27] Ng CS Yu SC Lau RW Yim AP. Hybrid DynaCT-guided electromagnetic navigational bronchoscopic biopsydagger. Eur J Cardiothorac Surg (2016) 49:i87–i8. doi: 10.1093/ejcts/ezv405 26604298

[28] Shi J He J He J Li S. Electromagnetic navigation-guided preoperative localization: the learning curve analysis. J Thorac Dis (2021) 13:4339–48. doi: 10.21037/jtd-21-490 PMC8339733 34422360

[29] Yuan HB Wang XY Sun JY Xie FF Zheng XX Tao GY. Flexible bronchoscopy-guided microwave ablation in peripheral porcine lung: a new minimally-invasive ablation. Transl Lung Cancer Res (2019) 8:787–96. doi: 10.21037/tlcr.2019.10.12 PMC6976383 32010557

